# Salivary Protein Profile and Food Intake: A Dietary Pattern Analysis

**DOI:** 10.1155/2021/6629951

**Published:** 2021-04-15

**Authors:** Teresa Louro, Carla Simões, Wilmara Lima, Laura Carreira, Paula Midori Castelo, Henrique Luis, Pedro Moreira, Elsa Lamy

**Affiliations:** ^1^MED (Mediterranean Institute for Agriculture, Environment and Development), Institute for Advanced Studies and Research (IIFA)IIFA–Instituto de Investigação e Formação Avançada, University of Évora, Évora, Portugal; ^2^Student from the Department of Chemistry, University of Évora, Évora, Portugal; ^3^Dept. Pharmaceutical Sciences, Universidade Federal de São Paulo (UNIFESP), Diadema, Brazil; ^4^School of Dental Medicine, University of Lisbon, Research Unit of Oral and Biomedical Sciences (UICOB), Lisbon, Portugal; ^5^Center for Innovative Care and Health Technology (ciThecCare), Politécnico de Leiria, Leiria, Portugal; ^6^Faculdade de Ciências da Nutrição e Alimentação da Universidade Do Porto, Porto 4150-180, Portugal

## Abstract

Saliva research has gained interest due to its potential as a source of biomarkers. One of the factors inducing changes in saliva, in the short term, is food intake, and evidence exist about changes in salivary proteome induced by some food components. Since this topic of research is in its early stages, it was hypothesized that saliva protein composition could be associated with different levels of adherence to dietary patterns that contain higher amounts of plant products. The aim of the present study was to test this hypothesis, in adults, by comparing salivary protein electrophoretic profiles of individuals with different diet characteristics, particularly dietary patterns (DP) that exhibit different proportions of animal and plant-based products. Dietary habits were assessed in 122 adults (61 from each sex, with ages ranging from 20 to 59 years) using Food Frequency Questionnaires. To identify the dietary patterns, a principal component analysis was used. Individual's non-stimulated saliva was evaluated for flow rate, pH, protein concentration, *α*-amylase activity, and electrophoretic protein profiles. Seven dietary patterns (DP) were identified. Salivary amylase enzymatic activity was positively associated with animal-based and starchy foods DP, and with plant-based fatty foods without wine DP. At the same time, protein bands containing amylase and type S cystatins were positively associated with the cheese/yoghurt and wine DP. Our results support the association of salivary proteomics and different dietary patterns and highlight the need of considering food consumption habits in studies using saliva, since this is a factor associated with variations in the composition of this fluid.

## 1. Introduction

Nowadays, due to global climate changes and the rise of food-related diseases (e.g., obesity, cardiovascular diseases, etc.), dietary patterns are becoming a major issue. Different methodologies are used to access dietary habits, most of which rely on subjective reports by individuals, which implies engagement and memory capacities [1], which may result in increased error in the data obtained. Moreover, individuals often misreport dietary intake, expressing what they believe to be adequate eating amounts, this misreport being even more frequent for people with a history of dieting and being overweight [2, 3]. For this reason, objective biomarkers identification continues to be important to increase accuracy of dietary intake assessment. Some biological markers have been tentatively assessed, with reports of urinary biomarkers of total protein [4], coffee and tea [5] or garlic intake [6], as well as plasmatic biomarkers of fruit and vegetable intake [7], among others, having been used.

Saliva study gained interest due to the potential of this fluid as a non-invasive and stress-free source of biomarkers for different pathologies and physiological conditions [8, 9]. In the nutrition field of study, the relationship between saliva and diet starts now to be studied (e.g., [10]) but many aspects remain to be understood. There are evidences that saliva participates in both oral processing and food sensory perception (e.g., [11–13]). Moreover, salivary gland secretion responds to diet, representing a potential source for intake biomarkers that needs to be explored. In animals, it was observed that species having different feeding niches differ in their salivary proteomes [14, 15]. Tannins (a type of polyphenols) are the major dietary compounds linked to the salivary differences that these animal species present, and it is possible that these differences are due to the tannin amounts in food. In fact, changes in salivary glands and in the amounts of particular salivary proteins (e.g., proline-rich proteins and alpha amylase) have been linked to the proportion of polyphenols/tannins in diet (e.g., [16–18]). From our knowledge, for humans, limited information exists about potential variations in saliva composition according to polyphenol intake levels, although the perception of astringency induced by these compounds has been demonstrated to be related to saliva composition [19]. Another nutritional compound potentially linked to saliva composition is starch. Variations in starch levels in diet was proposed to be related to differences in amylase gene (Amy1) copy number variants, with higher number of copies in populations with diets rich in starch [20, 21]. Additionally, short-term changes in salivary proteome were observed for bread ingestion [22, 23].

According to what was stated above, it is possible to hypothesize that saliva protein composition can vary among adults with different dietary patterns. Moreover, a relationship between saliva and diet composition was recently proposed, in studies performed in children [10, 24], reinforcing this hypothesis.

The objective of this work was to evaluate the potential of saliva as a non-invasive and objective tool for assessing potential association with dietary intake in adults, which may be of valuable use, particularly in new epidemiological studies.

## 2. Material and Methods

### 2.1. Participants

A convenience sample of 122 participants (61 from each sex), 20 to 59 years old, were recruited from North Alentejo region of Portugal, which is an interior region from the country characterized by a close link to agriculture. Participants were randomly recruited from all the municipalities of the referred region, since some variability in food habits may exist between cities and villages. This allowed us to have higher representativity of the region, although comparisons between dietary habits between different regions were not the aim of the present study. Only participants without self-reported and visible signs of oral (e.g., caries) or nasal health problems (e.g., smell loss, obstruction, etc.) participated in this study. On the data collection day, participants were asked to arrive at test room between 10:00 am. and 11:00 am., at least 1 h 30 m after breakfast intake. Before the beginning of the study, all subjects read and signed an informed consent form. All procedures were performed according to the Declaration of Helsinki for Medical Research Involving Human Subjects and had ethical approval from the Ethical Committee of the University of Evora.

### 2.2. Anthropometric Data Collection

Due to the potential association between Body Mass Index (BMI) and saliva composition [13, 25], height and weight were assessed and measured according to the European Health Examination Survey procedures [26]. Since the relationship between food intake and obesity was not a major aim of this study, only BMI was assessed (rather than other anthropometric measures) for controlling a possible effect in saliva composition. The participants were in a stand position wearing light cloths and barefoot. A portable stadiometer (Seca 214) and a digital scale accurate to the nearest 0.1 kg (Seca 803) were used to access height and weight, respectively. BMI was calculated by dividing the weight (kg) by the square of the height (m^2^). Normal weight, pre-obese, and obese were considered for the respective values: 18.5 < BMI < 25, 25 < BMI < 30, and BMI > 30 [27]. Since only one individual presented a BMI lower than 18.5, she was considered in normal weight group.

### 2.3. Food Intake Assessment

Participants were asked to report their frequency of food consumption by completing a self-administered, semi-quantitative food frequency questionnaire (FFQ), validated for Portuguese adults [28]. Before questionnaire completion, the researcher responsible for data collection explained how the questionnaires should be filled. The FFQ is an 86-item questionnaire, that includes individual food items and/or food groups and beverages and assesses usual dietary intake frequency over the previous 12 months. A blank space to introduce other foods, not correspondent to any of the 86 items, was provided. In that blank space, participants wrote the food name, portion, and intake frequency, using the same frequency possibilities. Food intake was calculated by multiply one of the nine possibilities of frequency of consumption (from “never or less than once per month” to “six or more times a day”), by the weight of the standard portion size of the food item, resulting in the daily intake of each food item. Participants were instructed to fill the questionnaire taking into consideration the food portion indicated. For example, if meat is consumed one time per day, in an amount representing the double of the portion indicated, then participant filled as consuming 2 times per day. Energy and nutritional intake were estimated using an adapted Portuguese version of the nutritional analysis software Food Processor Plus (ESHA Research Inc., Salem, OR, USA). When other foods or culinary food preparations, not contained in the 86 items of the FFQ, were reported, their nutritional value was estimated and added to capture the person's total nutrient intake.

For further constitution of dietary patterns, foods with similar nutritional characteristics were grouped, according to a previous study [29], resulting in 29 food/food groups that were further used in a multivariate analysis, as further described. The daily intake of each food group was obtained by summing the individual daily intakes of the foods that constituted it (for example, the daily intake of fresh fruit represents the sum of individual fruits daily intakes). Since that previous work was in children [29] and did not consider alcoholic beverages in that age group, we added wine and alcoholic beverages different from wine as two variables to include in the constitution of the dietary patterns.

### 2.4. Saliva Collection and Cleaning

Saliva collection occurred with a minimum of 1 h 30 m from the last food or beverage intake (breakfast), to avoid any influence of previous ingestion in salivary protein composition. Before saliva collection, individuals were asked to rinse with water to remove any food debris and “old” saliva. Saliva was collected without stimulation. Participants were requested to accumulate all saliva produced in the mouth and spitting it to a tube, maintained on ice, each time they need it. This collection occurred for a period of 3 minutes. Saliva was maintained on ice until laboratory arrival, where it was stored for one day at −20°C. To remove mucins and cell and/or food residues, saliva samples were thawed on ice and centrifuged at 13,000 *g* for 30 minutes at 4°C. The supernatant was recovered and stored at −80°C until subsequent analysis.

### 2.5. Saliva Composition Analysis

#### 2.5.1. Saliva Flow Rate, pH, and Total Protein Concentration

Saliva flow rate was assessed by assuming that saliva density is 1.0. Tubes containing saliva were weighed, with the weight of the empty tube being subtracted. The final value was divided by the number of minutes during which saliva was collected. The pH of saliva samples was measured using a calibrated pH meter (Hanna Instruments) and recording to two decimal places. Total protein concentration was determined by the Bradford method, using bovine serum albumin (BSA) as standard, and plates were read at 600 nm in a microplate reader (Glomax, Promega).

#### 2.5.2. SDS-PAGE Separation and Protein Profile Analysis

Each saliva sample was run in duplicate. For each sample, a volume corresponding to 7.5 *μ*g total protein was mixed with sample buffer and run on each lane of a 14% polyacrylamide mini-gel (Protean xi, Bio-Rad, CA, USA) using a Laemmli buffer system, as described elsewhere [30]. Each electrophoretic run was performed at a constant voltage of 140 V until front dye reached the end of the gel. Gels were fixed for 1 hour in 40% methanol/10% acetic acid, followed by staining for 2 hours with Coomassie Brilliant Blue (CBB) G-250. Gel images were acquired using a scanning Molecular Dynamics densitometer with internal calibration and LabScan software (GE Healthcare), and images were analysed using GelAnalyzer software (GelAnalyzer 2010a by Istvan Lazar, http://www.gelanalyzer.com) for the volume percentage of each protein band. Molecular masses were determined in accordance with molecular mass standards (Bio-Rad Precision Plus Protein Dual Colour 161–0394) run with protein samples. The identification of the proteins contained in the bands observed in SDS-PAGE salivary profiles was based on previous data [22].

#### 2.5.3. Salivary Amylase Enzymatic Activity

A Salimetrics® kit was used to determine the enzymatic activity of salivary amylase according to the manufacturer's recommendations. Briefly, saliva samples were diluted 200× and applied on the microplate in duplicate, followed by application of a substrate (2-chloro-p-nitrophenol) preheated to 37°C. The mixture was incubated at 37°C for 1 minute, and absorbance values were read at 405 nm in a plate reader spectrophotometer, followed by incubation for an additional 2 minutes at 37°C and a new reading at 405 nm. The enzymatic activity of amylase (U/ml) was calculated by the following formula: (ΔAbs./min × TV × DF)/(MMA × SV × LP), where ΔAbs./Min is absorbance variation per minute, TV is total test volume (0.287 mL), DF is dilution factor, MMA is millimolar absorbance of substrate 2-chloro-p-nitrophenol (12.9), SV is sample volume (0.007 mL), and LP is light path (0.97, specific for plate received with kit).

### 2.6. Statistical Analysis

The values of total protein concentration, salivary secretion rate, protein band amount (volume percentage), and salivary amylase enzymatic activity were analysed statistically. Descriptive statistics was performed, and normality and homoscedasticity were tested through Shapiro–Wilk and Levene tests, respectively. Continuous variables were compared between sexes using Student's *t*-test when assumptions were fulfilled and using Mann–Whitney when not. For BMI classes (normal weigh, pre-obese and obese) comparison, one-way ANOVA or the non-parametric Kruskal–Wallis test was used, considering a confidence interval of 95%.

To identify dietary patterns within the study population, multivariate statistical techniques were used. The 29 foods/food groups constituted from the 86 items of the FFQ, as described earlier and according to a previous study [29], were considered together with 2 additional groups, namely, wine, and alcoholic beverages excluding wine. These 31 variables (amount of food consumed per day for each food/food group) were reduced through principal component analysis (PCA). PCA with orthogonal rotation (Varimax) was used to estimate the latent factors emerging and to obtain optimal non-correlated components. The number of components (dietary patterns) to be retained was decided based on the observation of correlation matrix, on the total of variance explained by the components and on Kaiser–Meyer–Olkin (KMO) measure of sampling adequacy values above 0.600 [31, 32].

With the aim of explaining *α*-amylase enzymatic activity and salivary protein profile variation (namely, the expression levels of each SDS-PAGE protein band) as dependent variables, each dietary pattern extracted from the PCA analysis was used as independent variable in the regression model, adjusting for age, BMI, sex, and total energy consumed. A stepwise backward process was used to obtain the final model after looking for changes in adjusted *R*2 and *F* values for each retained variable; the assumptions of collinearity (VIF and tolerance), non-dependent errors (Durbin–Watson), and homoscedasticity (residual analysis) were also considered.

## 3. Results

### 3.1. Participants Characteristics and Dietary Patterns

Forty percent of the individuals participating in this study were normal weight and 60% overweight (36% pre-obese + 24% obese). When comparing the sexes, men presented statistically significantly higher BMI than women (Table 1), with 67.7% of men being overweight (38.7% pre-obese + 29.0% obese) whereas 29.8% of women being pre-obese and 17.5% obese (e.g., a total of 47.3% overweight women).

When participants were analysed for their food intake, assessed by the FFQ, some statistically significant differences were observed between men and women in the energy contribution of protein (higher in women) and in the consumption of some food groups (Table 1). Women have significant higher consumption of vegetables, vegetable soup, yoghurts, and crackers/cookies with approximately 20% sugar or less, as well as white meat and lower consumption levels of canned fruit, red meat, processed meat, and alcoholic drinks. No significant differences between sexes were found in relation to mean total energy or total macronutrient intake.

It was observed a tendency for older individuals to have higher BMI. Despite no significant differences in total energy or total macronutrient intake, for foods like vegetable soup, vegetable oil, and starchy-rich foods, the daily intake was observed to be higher in obese individuals (Table 1).

To estimate the dietary patterns from the food/food groups considered [29], PCA was performed as described in material and methods section. PCA adequacy was evaluated before analysis. Matrix component inspection allowed choosing the variables (food/food groups) with coefficients higher than 0.30 in at least one component, resulting in 24 variables (food/food groups) including the final model. The general measure of KMO was 0.606, and Bartlett sphericity was statistically significant (*p* < 0.0001).

It was possible to obtain 7 components, which explained 59% of total variance (Table 2), corresponding to the following dietary patterns: DP1 with positive loadings for vegetables and soup, olives, fresh fruit, bread and starch-rich foods, and sugar sweetened beverages (SSB) (plant-based with SSB DP); DP2 presented positive loadings for fish, meat, eggs, and starch-rich foods (animal-based and starchy foods pattern); DP3 presented high positive scores for fast food, processed foods, and SSB (fast-food pattern); DP4 presented positive loadings for olive oil, margarine, and butter, as well as for pastry and negative loadings for pulses (dietary fats and pastry pattern); DP5 presented positive loadings for dairy desserts, pastry, canned fruits, and ready-to-eat cereals (sweet foods pattern); DP6 presented positive loadings for olives and nuts, but negative loadings for wine (plant-based fatty foods without wine pattern); and DP7 presented positive loadings for yoghurt, cheese, and wine (cheese/yoghurt and wine pattern).

### 3.2. Saliva Composition

**Figure 1:**
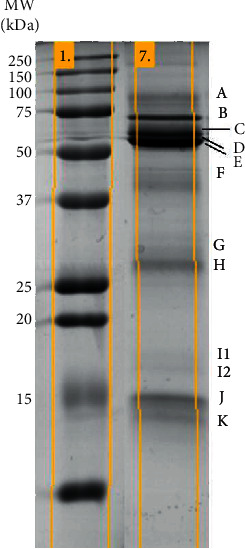
Representative SDS-PAGE salivary profile showing the 12 protein bands consistently present in the different individuals studied (letters on the right side); MW, molecular mass (kDa).

SDS-PAGE electrophoresis allowed the consistent separation and comparison of well resolved 12 protein bands, with apparent molecular masses between 14.1 and 88.0 kDa (Figure 1), containing proteins previously identified [22] (Table 3).

By comparing the sexes for saliva composition, men presented higher expression levels of the protein bands G (4.39 ± 0.73 vs. 3.98 ± 0.85, for men and women, respectively; *p* = .005) and J (8.54 ± 2.74 vs. 7.41 ± 2.14, for men and women, respectively; *p* = .014).

### 3.3. Dietary Patterns and Saliva Composition

The significant associations found for linear multiple regression models between salivary parameters, namely, amylase enzymatic activity, protein bands E (identified as amylase) and J (identified as cystatins), and dietary patterns, are shown in Table 4. The protein bands A, B, I1, I2, and K did not show significant associations, and protein bands C, D, and G were only associated with age (positively), BMI (positively), and sex (high in men), respectively.

Looking at the linear multiple regression models between dietary patterns and salivary parameters, adjusting for confounders (sex, age, BMI, and energy intake), in Table 4, it was possible to observe that DP7 (fermented dairy and wine pattern) was positively associated with bands E (containing amylase) and J (containing S-type cystatins), and DP6 (plant-based fatty foods without wine pattern) and DP2 (animal-based and starchy foods pattern) were positively associated with amylase enzymatic activity.

## 4. Discussion

In the present study, PCA analysis was used to identify combinations of foods, named as dietary patterns and which constituted not-correlated variables, used as independent variables in the regression models. As such, these dietary patterns do not represent groups of individuals with particular food consumption patterns.

Dietary patterns constituted by high proportion of plant-based fatty foods (without wine, DP6) together with dietary patterns based on foods of animal origin and starch-rich foods (DP2) were associated with the salivary amylase enzymatic activity. This is in line with other studies associating salivary amylase with starchy foods intake [21] although, to the best of our knowledge, no previous studies related salivary amylase with olives and nuts intake. The different results obtained when amylase was considered in terms expression levels, comparatively to when it was considered in terms of its enzymatic activity, are not surprising, since a lack of association between the enzymatic activity of this protein and the expression levels of its different proteoforms has been previously reported [33].

In relation to a dietary pattern with high contribution of fermented dairy and wine (DP7), an association with the expression level of one band containing *α*-amylase and one band containing S-type cystatins was observed. Concerning wine, it may be a source of tannins, which are known to affect saliva proteome, in animal models [16, 34]. In fact, in mice, it was previously observed that increased levels of polyphenols (tannins) in the diet result in increased expression levels of salivary amylase bands, in SDS-PAGE profiles [18]. Concerning cystatins, these are inhibitors of cysteine proteases, the family of S-type cystatins being secreted by salivary glands. Different studies show a relationship between salivary cystatins and the bitterness or astringency (e.g., [13, 35, 36]) that is associated with the polyphenol content of foods and beverages, such as wines (particularly red wines). If higher levels of salivary S-type cystatins are present in individuals with low sensitivity to bitterness and astringency, as reported by some authors [11], it can be hypothesized that the positive association between S-type cystatins and DP7 results from the lower intensity with which these oral sensations are perceived and consequently the higher acceptance/preference for wine, in consequence of salivary cystatins. At the same time, another hypothesis is that a higher intake of wine can induce higher levels of salivary S-type cystatins. This hypothesis is supported by studies showing that exposing rats to tannins or bitter compounds results in the increase of S-type salivary cystatins secretion and in the increase of bitter taste compounds acceptance [35, 37]. Anyway, this hypothesis needs to be further tested. It is worthwhile to remember that DP7 has also a considerable contribution of fermented dairy. Although few information exists about effect of dairy in saliva composition, the levels of salivary cystatins were recently reported to increase in response to chocolate milk [38].

The association between the dietary patterns obtained by PCA analysis and saliva composition may be additionally confounded by sex, BMI, and age. As such, those parameters were included in the regression models used. Although we are unable to guarantee that the observed salivary differences between sexes are due exclusively to the sex factor, and not to the differences in the eating habits of women and men, the effect of sex in salivary proteome needs to be considered, since it has been already reported [13, 39, 40] and reinforced in recent publications (e.g., [41]). Minor differences were observed among BMI groups in food consumption habits, although starchy foods were highly consumed by overweight and obese individuals. As for the case of sex, we cannot guarantee that the effect of BMI in saliva was not influenced by this particular difference in food intake. As such, the positive association between BMI and the band containing one amylase form (band E) can result from this higher intake of starchy foods by individuals with high BMI. But, since band E was not related to the patterns with starch-rich foods (DP2), other factors apart from starch consumption may explain this positive association between band E and obesity. Differences in saliva composition among individuals with different BMI have been already observed, which included variations in the expression levels of salivary proteins like carbonic anhydrase VI and some forms of amylase [30]. Finally, the positive association between salivary amylase enzymatic activity and age goes in line with previous observations about increasing salivary amylolytic activity in people with higher age [42].

### 4.1. Our Study Has Some Limitations

1. Firstly, although in the regression model for statistical analysis, *α*-amylase enzymatic activity and salivary protein profile were considered as dependent variables, while dietary patterns extracted from the PCA analysis were used as independent variables, the cross-sectional design of our study does not allow us to establish causal relationships between dietary intake and salivary parameters. Moreover, since the dietary patterns considered are, in fact, the components generated by PCA, which consist in the combinations of the different food/food groups, it is not possible to allocate each individual subject to one of these dietary patterns.
2. Secondly, whereas the salivary parameters represent the saliva collected at one time point, the intake reported represents the frequency of the food groups consumption in the last 12 months. Short-term effect of food intake was not controlled. Moreover, it is recognized that FFQ have the limitation of being difficult to carry out to complete, and suffer from difficulties in self-estimation of portion size and biases resulting from misreporting [43]. However, the FFQ used is validated for Portuguese adults and considered as a valid tool to estimate the usual food intake in large samples. Intervention-based studies must be further done to get more controlled results about the effect of specific dietary patterns in saliva composition.

Our study has also important strengths. According to our best knowledge, this is the first study that aimed to assess how dietary habits relate to saliva protein composition. One of the main findings is that some types of salivary proteins appear to be associated with the intake of particular foods, namely, animal-based and starch-rich foods, plant-based fatty foods, fermented dairy, and wine. It is a fact that the regression models obtained presented relatively low *R*^2^, which means that the model only explains a minor percentage of the variation in salivary proteins levels. But taking into consideration that we are working with biological models, where several other factors besides food frequency consumption can affect salivary protein composition [44, 45], this low *R*^2^ is not surprising and the statistical significance of the model reinforces an association between salivary proteins and food habits.

## 5. Conclusions

The results obtained in the present study emphasize the potential of saliva to reflect differences in food intake. Salivary amylase and S-type cystatins are associated with dietary patterns. It is interesting to note that some combinations of foods are more related to the total enzymatic activity of amylase whereas others with the expression levels of forms of this protein. Salivary amylase and cystatins are proteins previously observed to have a link to oral food perception and more studies are necessary to confirm if they can be the result of the type of food eaten or rather the cause of food choices.

To consider salivary proteins as non-invasive biomarkers of intake, more studies are required, to define criteria, such as sex, BMI, age, and hour of the day, among others. Moreover, it will be important, in further work, to get more detailed information using other proteomic approaches. Even so, this study evidences that some of the variation in salivary protein composition, among individuals, can be related to food habits and it is important to take into account foods as a source of variation, when using saliva for studies with different purposes.

## Figures and Tables

**Table 1:** Sample characteristics, nutritional, and food intake differences between sexes and BMI (values are mean ± standard deviation (SD)).

Parameters	Men	Women	*p*-value (sex)		NW	Pre-obese	Obese	*p*-value (BMI)
Age (years)	40.7 ± 10.9	38.3 ± 10.7	0.477		37.1 ± 10.9	41.5 ± 1.7	42.6 ± 9.6	.054
BMI (kg/m^2^)	27.9 ± 4.2	25.9 ± 5.2	0.014 ^*∗*^		—	—	—	

Nutritional parameters								
Energy (kcal/day)	2346 ± 746	2280 ± 734	0.627		2304 ± 723	2361 ± 787	2211 ± 538	0.650
Protein (g/day)	102.8 ± 37.6	107.8 ± 35.6	0.457		103.1 ± 36.1	105.5 ± 35.7	106.5 ± 32.1	0.924
Protein (% TEV)	17.6 ± 3.9	19.2 ± 3.7	0.027 ^*∗*^		18.1 ± 4.0	18.1 ± 3.3	19.4 ± 4.3	0.198
Total carbohydrate (g/day)	263.4 ± 101.7	256.1 ± 92.2	0.911		265.3 ± 95.0	268.4 ± 106.5	236.8 ± 75.0	0.472
Total carbohydrate (% TEV)	44.9 ± 10.0	44.9 ± 8.8	0.976		46.3 ± 11.0	45.4 ± 7.0	42.4 ± 8.1	0.143
Total sugars (g/day)	119.0 ± 58.5	113.5 ± 51.4	0.853		124.9 ± 60.5	111.5 ± 47.7	103.6 ± 44.6	0.351
Total sugars (% TEV)	20.5 ± 7.8	19.9 ± 6.4	0.651		21.6 ± 8.6	19.4 ± 4.9	18.5 ± 5.9	0.226
SFA (g/day)	26.6 ± 11.0	26.3 ± 9.5	0.951		26.7 ± 10.1	26.8 ± 11.4	25.6 ± 8.9	0.866
SFA (% TEV)	10.3 ± 2.6	10.5 ± 2.3	0.684		10.5 ± 2.5	10.2 ± 2.4	10.5 ± 2.2	0.949
MUFA (g/day)	41.2 ± 16.9	42.9 ± 17.8	0.535		40.2 ± 16.3	43.0 ± 17.9	41.4 ± 13.9	0.685
MUFA (% TEV)	15.8 ± 4.0	17.1 ± 4.0	0.072		16.0 ± 4.0	16.3 ± 3.9	16.8 ± 3.3	0.458
PUFA (g/day)	15.8 ± 6.0	15.8 ± 6.4	0.901		14.6 ± 5.7	16.6 ± 6.2	15.9 ± 5.8	0.137
PUFA (% TEV)	6.1 ± 1.5	6.3 ± 1.4	0.351		5.9 ± 1.5	6.3 ± 1.3	6.4 ± 1.3	0.106
Cholesterol (mg/day)	361.3 ± 167.7	345.6 ± 134.0	0.578		345.0 ± 148.3	358.6 ± 165.9	369.3 ± 141.7	0.774
Fibre (g/day)	26.2 ± 13.0	28.4 ± 12.9	0.206		27.8 ± 13.0	28.5 ± 13.4	25.7 ± 12.5	0.695

Foods/food groups (g/day) ^#^								
Vegetables	387.9 ± 268.0	499.7 ± 255.4		0.038 ^*∗*^	434.9 ± 283.1	424.4 ± 230.6	543.4 ± 265.5	0.106
Vegetable soup	194.5 ± 167.4	272.0 ± 154.6		0.003 ^*∗*^	247.6 ± 237.2	206.0 ± 130.1	298.0 ± 136.9	0.016 ^*∗*^
Fresh fruits	253.8 ± 177.1	353.6 ± 239.3		0.027 ^*∗*^	348.6 ± 270.3	304.6 ± 196.8	255.1 ± 188.3	0.281
Canned fruits	10.7 ± 21.2	5.6 ± 12.8		0.006 ^*∗*^	6.4 ± 13.5	9.3 ± 23.5	7.8 ± 11.8	0.300
Olives	8.1 ± 15.4	13.4 ± 36.6		0.230	14.3 ± 38.9	8.9 ± 18.2	7.7 ± 12.3	0.719
Nuts	10.6 ± 15.0	17.2 ± 22.9		0.193	13.9 ± 29.9	15.5 ± 19.3	17.2 ± 23.2	0.223
Fish	72.7 ± 46.3	82.9 ± 6.4		0.168	68.5 ± 43.6	82.7 ± 48.2	89.6 ± 55.6	0.156
White meat	55.1 ± 47.0	72.9 ± 47.7		0.039 ^*∗*^				
Red meat	43.0 ± 34.3	30.8 ± 27.3		0.032 ^*∗*^	32.7 ± 27.8	38.1 ± 35.4	38.8 ± 27.9	0.572
Processed meat^1^	31.9 ± 40.5	17.5 ± 14.3		0.012 ^*∗*^	23.8 ± 34.4	24.5 ± 35.0	24.6 ± 17.1	0.347
Eggs	14.1 ± 11.1	14.7 ± 9.8		0.397	12.9 ± 9.5	15.4 ± 11.6	15.7 ± 10.5	0.325
Olive oil	10.9 ± 9.5	12.9 ± 9.1		0.084	11.4 ± 10.6	14.0 ± 12.9	11.6 ± 9.1	0.868
Vegetable oil	2.1 ± 3.5	1.5 ± 3.1		0.213	1.0 ± 2.4	2.3 ± 3.3	2.0 ± 3.8	0.006 ^*∗*^
Butter	2.5 ± 2.2	3.5 ± 3.0		0.059	2.9 ± 2.3	2.9 ± 2.7	2.9 ± 3.1	0.843
Margarine	1.2 ± 1.6	1.3 ± 1.6		0.438	1.7 ± 2.2	1.5 ± 2.3	1.2 ± 2.4	0.366
Milk	167.7 ± 164.4	231.5 ± 206.7		0.114	199.4 ± 212.4	216.4 ± 179.4	186.9 ± 152.5	0.524
Yoghurt	62.7 ± 85.3	93.6 ± 90.6		0.009 ^*∗*^	84.6 ± 99.9	60.4 ± 72.6	86.0 ± 90.9	0.399
Cheese	18.1 ± 21.3	15.7 ± 16.3		0.889	16.7 ± 19.3	16.5 ± 19.2	19.0 ± 19.2	0.623
Milk-based puddings	13.9 ± 16.6	9.2 ± 9.2		0.323	11.1 ± 13.2	12.0 ± 15.3	10.2 ± 10.7	0.982
Ice-cream	7.5 ± 8.7	5.6 ± 8.1		0.083	6.3 ± 8.4	7.9 ± 10.4	4.9 ± 4.7	0.791
Starch-rich foods (rice, potatoes, pasta)^2^	136.8 ± 72.0	136.4 ± 71.1		1.000	120.1 ± 66.7	156.5 ± 53.8	153.8 ± 109.4	0.021 ^*∗*^
Bread	63.8 ± 51.7	77.0 ± 53.7		0.110	76.6 ± 54.7	75.1 ± 57.1	59.4 ± 39.7	0.542
Ready-to-eat cereals	11.6 ± 14.8	9.3 ± 14.1		0.306	9.5 ± 14.2	12.5 ± 15.3	7.5 ± 12.9	0.357
Sugar sweetened beverages (SSB)^3^	170.1 ± 216.3	84.6 ± 100.2		0.100	134.6 ± 192.4	107.2 ± 145.3	128.1 ± 180.6	0.839
Coffee	76.5 ± 57.6	63.4 ± 49.5		0.330	64.5 ± 56.6	74.5 ± 49.5	78.8 ± 50.7	0.585
Tea	32.4 ± 58.6	68.3 ± 88.3		0.246	42.2 ± 79.7	45.2 ± 74.0	43.1 ± 72.1	0.994
Fast-food^4^	37.8 ± 27.8	29.3 ± 15.4		0.163	32.7 ± 23.8	32.1 ± 20.3	37.5 ± 33.8	0.936
Pastry	40.1 ± 36.4	30.6 ± 25.7		0.245	36.0 ± 31.0	40.3 ± 38.1	25.7 ± 22.7	0.237
Crackers/cookies^5^	5.5 ± 7.6	10.9 ± 13.5		0.029 ^*∗*^	9.8 ± 12.4	7.7 ± 10.4	6.8 ± 9.9	0.433
Pulses	51.9 ± 57.6	35.5 ± 36.7		0.498	44.2 ± 49.9	46.6 ± 50.1	42.9 ± 45.6	0.975
Wine	36.6 ± 41.3	12.2 ± 25.3		<0.001 ^*∗*^	17.4 ± 30.8	25.4 ± 36.9	38.0 ± 42.6	0.072
Alcoholic beverages (apart from wine)^6^	61.9 ± 74.5	14.1 ± 30.4		<0.001 ^*∗*^	26.1 ± 43.6	43.1 ± 63.2	52.1 ± 83.6	0.173

^#^The food/food groups according to previous work [29], which were further used to constitute the dietary patterns.^1^Processed meats include ham, sausages, and bacon; ^2^Starch-rich foods include pasta, rice, and potato. ^3^Sugar sweetened beverages include all types of juices with sugar, cola, and ice-tea. ^4^Fast-food includes hamburger, pizza, snacked fried food, and sausages. ^5^Cookies with sugar amounts lower that 20% of total ingredients. ^6^Beer + spirits.  ^*∗*^*p* < .05. SFA, saturated fatty acids; MUFA, monounsaturated fatty acids; PUFA, polyunsaturated fatty acids; TEV, total energy value.

**Table 2:** Component loadings obtained by principal component analysis with Varimax rotation. Each component was named as “dietary pattern.”

% Var explained	Component						
	1	2	3	4	5	6	7
	16.0	11.0	8.7	6.9	5.7	5.4	5.0
Yoghurt							0.422
Cheese							0.741
Milk-based puddings					0.599		
Eggs		0.637					
Meat		0.765					
Processed meat			0.575				
Fish		0.823					
Olive oil				0.665			
Margarine				0.633			
Butter				0.740			
Bread	0.779						
Ready-to-eat cereals					0.584		
Starch-rich foods (rice, potatoes, pasta)	0.456	0.471					
Pastry				0.486	0.472		
Vegetables	0.725						
Pulses				−0.418			
Fresh fruits	0.521						
Canned fruits					0.727		
Nuts						0.732	
Olives	0.449					0.667	
SSB^1^	0.467		0.637				
Fast-food			0.778				
Vegetable soup	0.712						
Wine						−0.518	0.423

Rotation converted with 16 interactions. Values lower than 0.40 were omitted. ^1^Sugar sweetened beverages.

**Table 3:** Proteins present in the bands observed in SDS-PAGE salivary profiles.

SDS-PAGE Band	Protein identification#	Apparent molecular mass (kDa)
A	Polymeric immunoglobulin receptor	88.0
B	Serum albumin	71.0
C	*α*-Amylase 1	64.0
D	*α*-Amylase 1	58.2
E	*α*-Amylase 1	55.0
F	Zinc-*α*2-glycoprotein	42.0
	Carbonic anhydrase VI	
G	Zymogen granule protein 16 homolog B	31.5
	Immunoglobulin kappa constant	
H	Immunoglobulin kappa constant	27.0
I1	Prolactin inducible protein (PIP)	16.8
I2	Prolactin inducible protein (PIP)	16.2
J	Cystatin-SN	14.5
	Cystatin-S	
K	Cystatin B	14.1

^#^Protein identification based on previous studies with human saliva collected in the same conditions.

**Table 4:** Linear multiple regression models between dietary patterns and salivary parameters.

Dependent variable	Independent variable	Coeff	CI (95%)	*t*	*p* value	*F*	*R*2 adj	Durbin− Watson
Band E	Constant	1.390	0.686 to 2.095	3.912	<0.001	2.765	0.072	1.953
	Dietary pattern 7	0.106	0.012 to 0.199	2.247	**0.027** ^*∗*^			
	Energy	6.208E−6	0.000 to 0.000	0.136	0.892			
	Sex	0.023	−0.159 to 0.204	0.248	0.804			
	Age	−0.008	−0.017 to 0.000	−1.868	0.064			
	BMI	0.027	0.008 to 0.047	2.766	**0.007** ^*∗*^			

Band J	Constant	9.035	5.472 to 12.598	5.027	<0.001	3.868	0.132	1.598
	Dietary pattern 7	.691	0.224 to 1.157	2.934	**0.004** ^*∗*^			
	Energy	0.000	−0.001 to 0.000	−0.513	0.609			
	Sex	−0.625	−1.574 to .323	−1.307	0.194			
	Age	0.041	−0.002 to 0.085	1.892	0.061			
	BMI	−0.054	−0.151 to 0.043	−1.099	0.274			

*α*-Amylase (U/mL)	Constant	167.348	−46.456 to 381.152	1.553	0.124	4.126	0.150	1.569
	Dietary pattern 2	42.668	13.620 to 71.715	2.914	**0.004** ^*∗*^			
	Dietary pattern 6	31.012	2.395 to 59.629	2.150	**0.034** ^*∗*^			
	Energy	−0.007	−0.037 to 0.023	−.459	0.647			
	Sex	18.932	−36.866 to 74.730	0.673	0.502			
	Age	4.474	1.905 to 7.043	3.456	**0.001** ^*∗*^			
	BMI	−5.641	−11.433 to 0.150	−1.933	0.056			

All the models were adjusted for sex, age, BMI, and energy intake.  ^*∗*^Significant for *p* < 0.05.

## Data Availability

All the data necessary to reach the objectives of the study are included. Raw data files, which generated the results, are with the researchers.

## References

[1] Shim J.-S., Oh K., Kim H. C. (2014). Dietary assessment methods in epidemiologic studies. *Epidemiology and Health*.

[2] Maurer J., Taren D. L., Teixeira P. J. (2006). The psychosocial and behavioral characteristics related to energy misreporting. *Nutrition Reviews*.

[3] Johnson R. K., Soultanakis R. P., Matthews D. E. (1998). Literacy and body fatness are associated with underreporting of energy intake in US low-income women using the multiple-pass 24-hour recall: a doubly labeled water study. *Journal of the American Dietetic Association*.

[4] Bingham S. A. (2003). Urine nitrogen as a biomarker for the validation of dietary protein intake. *Journal of Nutrition*.

[5] Rothwell J. A., Madrid-Gambin F., Garcia-Aloy M. (2018). Biomarkers of intake for coffee, tea, and sweetened beverages. *Genes & Nutrition*.

[6] Scheffler L., Sauermann Y., Heinlein A., Sharapa C., Buettner A. (2016). Detection of volatile metabolites derived from garlic (Allium sativum) in human urine. *Metabolites*.

[7] Pennant M., Steur M., Moore C., Butterworth A., Johnson L. (2013). Comparative validity of Vitamin C and carotenoids as indicators of fruit and vegetable intake: a systematic review and meta-analysis of randomised controlled trials. *British Journal of Nutrition*.

[8] Lamy E. (2018). Salivary proteomics in ingestive behaviour research: advances, potentialities and limitations. *Journal of Integrated OMICS*.

[9] Castagnola M., Scarano E., Passali G. C. (2017). Salivary biomarkers and proteomics: future diagnostic and clinical utilities. *ACTA Otorhinolaryngologica Italica*.

[10] Morzel M., Truntzer C., Neyraud E. (2017). Associations between food consumption patterns and saliva composition: specificities of eating difficulties children. *Physiology & Behavior*.

[11] Dsamou M., Palicki O., Septier C. (2012). Salivary protein profiles and sensitivity to the bitter taste of caffeine. *Chemical Senses*.

[12] Cabras T., Melis M., Castagnola M. (2012). Responsiveness to 6-n-propylthiouracil (PROP) is associated with salivary levels of two specific basic proline-rich proteins in humans. *PLoS One*.

[13] Rodrigues L., da Costa G., Cordeiro C., Pinheiro C. C., Amado F., Lamy E. (2017). Relationship between saliva protein composition and 6-n-Propylthiouracil bitter taste responsiveness in young adults. *Journal of Sensory Studies*.

[14] Lamy E., da Costa G., Santos R. (2009). Sheep and goat saliva proteome analysis: a useful tool for ingestive behavior research?. *Physiology & Behavior*.

[15] Sales Baptista E., Lamy E., Mau M., Capela e Silva F., Coelho A. V. (2013). Variation in salivary protein composition related to feeding behavior and its ecological implications.

[16] Lamy E., Graça G., da Costa G. (2010). Changes in mouse whole saliva soluble proteome induced by tannin-enriched diet. *Proteome Science*.

[17] Lamy E., Baptista E. S., Coelho A. V., Silva F. C. (2010). Morphological alterations in salivary glands of mice (*Mus musculus*) submitted to tannin enriched diets: comparison with sialotrophic effects of sympathetic agonists stimulation. *The Arquivo Brasileiro de Medicina Veterinária e Zootecnia*.

[18] da Costa G., Lamy E., Capela e Silva F., Andersen J., Sales Baptista E., V Coelho A. (2008). Salivary amylase induction by tannin-enriched diets as a possible countermeasure against tannins. *Journal of Chemical Ecology*.

[19] Dinnella C., Recchia A., Vincenzi S., Tuorila H., Monteleone E. (2010). Temporary modification of salivary protein profile and individual responses to repeated phenolic astringent stimuli. *Chemical Senses*.

[20] Mandel A. L., Peyrot des Gachons C., Plank K. L., Alarcon S., Breslin P. A. S. (2010). Individual differences in AMY1 gene copy number, salivary *α*-amylase levels, and the perception of oral starch. *PLoS One*.

[21] Santos J. L., Saus E., V Smalley S. (2012). Copy number polymorphism of the salivary amylase gene: implications in human nutrition research. *Journal of Nutrigenetics and Nutrigenomics*.

[22] Carreira L., Midori Castelo P., Simões C., Capela e Silva F., Viegas C., Lamy E. (2020). Changes in salivary proteome in response to bread odour. *Nutrients*.

[23] Lamy E., Santos V., Barrambana S. (2020). *Saliva Protein Composition Relates with Interindividual Variations in Bread Sensory Ratings, Starch - Stärke*.

[24] Tvarijonaviciute A., Martinez-Lozano N., Rios R., Marcilla de Teruel M. C., Garaulet M., Cerón J. J. (2019). Saliva as a non-invasive tool for assessment of metabolic and inflammatory biomarkers in children. *Clinical Nutrition*.

[25] Rodrigues L., Espanca R., Costa A. R. (2019). Comparison of salivary proteome of children with different sensitivities for bitter and sweet tastes: association with body mass index. *International Journal of Obesity*.

[26] Nunes B., Barreto M., Gil A. P. (2019). The first Portuguese National Health Examination Survey (2015): design, planning and implementation. *Journal of Public Health*.

[27] WHO (2000). Preventing and Managing the Global Epidemic. World Health Organization: Technical Report Series.

[28] Lopes C., Aro A., Azevedo A., Ramos E., Barros H. (2007). Intake and adipose tissue composition of fatty acids and risk of myocardial infarction in a male Portuguese community sample. *Journal of the American Dietetic Association*.

[29] Moreira P., Santos S., Padrão P. (2010). Food patterns according to Sociodemographics, physical activity, sleeping and obesity in Portuguese children. *International Journal of Environmental Research and Public Health*.

[30] Lamy E., Simões C., Rodrigues L. (2015). Changes in the salivary protein profile of morbidly obese women either previously subjected to bariatric surgery or not. *Journal of Physiology and Biochemistry*.

[31] Kaiser H. F. (1974). An index of factorial simplicity. *Psychometrika*.

[32] Kaiser H. F. (1960). The application of electronic computers to factor Analysis. *Educational and Psychological Measurement*.

[33] Contreras-Aguilar M. D., Escribano D., Martínez-Subiela S., Martínez-Miró S., Cerón J. J., Tecles F. (2018). Changes in alpha-amylase activity, concentration and isoforms in pigs after an experimental acute stress model: an exploratory study. *BMC Veterinary Research*.

[34] Lamy E., Rawel H., Schweigert F. J. (2011). The effect of tannins on mediterranean ruminant ingestive behavior: the role of the oral cavity. *Molecules*.

[35] Martin L. E., Nikonova L. V., Kay K., Paedae A. B., Contreras R. J., Torregrossa A. M. (2018). Salivary proteins alter taste-guided behaviors and taste nerve signaling in rat. *Physiology & Behavior*.

[36] Soares S., Mateus N., de Freitas V. (2012). Interaction of different classes of salivary proteins with food tannins. *Food Research International*.

[37] Martin L. E., Nikonova L. V., Kay K. E., Torregrossa A. M. (2019). Altering salivary protein profile can increase acceptance of a novel bitter diet. *Appetite*.

[38] Crawford C. R., Running C. A. (2020). Addition of chocolate milk to diet corresponds to protein concentration changes in human saliva. *Physiology & Behavior*.

[39] Rodrigues L., Espanca R., Costa A. R. (2017). Association between salivary leptin levels and taste perception in children. *Journal of Nutrition and Metabolism*.

[40] Rodrigues L., Costa G., Cordeiro C., Pinheiro C., Amado F., Lamy E. (2017). Salivary proteome and glucose levels are related with sweet taste sensitivity in young adults. *Food & Nutrition Research*.

[41] Franco-Martínez L., González-Hernández J. M., Horvatić A. (2020). Differences on salivary proteome at rest and in response to an acute exercise in men and women: a pilot study. *Journal of Proteomics*.

[42] Mosca A. C., Stieger M., Neyraud E., Brignot H., van de Wiel A., Chen J. (2019). How are macronutrient intake, BMI, ethnicity, age, and gender related to the composition of unstimulated saliva? a case study. *Journal of Texture Studies*.

[43] Skinner A., Toumpakari Z., Stone C., Johnson L. (2020). Future directions for integrative objective assessment of eating using wearable sensing Technology. *Frontiers in Nutrition*.

[44] Esteves C. V., de Campos W. G., de Souza M. M., Lourenço S. V., Siqueira W. L., Lemos-Júnior C. A. (2019). Diagnostic potential of saliva proteome analysis: a review and guide to clinical practice. *Brazilian Oral Research*.

[45] Amado F. M. L., Ferreira R. P., Vitorino R. (2013). One decade of salivary proteomics: current approaches and outstanding challenges. *Clinical Biochemistry*.

